# Supplementary material for: Geometry‐Based Neural‐Network Prediction of Electron Localization Function Topology in Dense Hydrogen
Source: Chemistry. 2026 Jul 6;32(39):e00030. doi: 10.1002/chem.202600030 (PMC13715478; doi:10.1002/chem.202600030)
Supplement: Supplementary file 1 [file CHEM-32-e00030-s001.pdf]

# Supporting Information for Geometry-Based Neural-Network Prediction of Electron Localization Function Topology in Dense Hydrogen

Xiaoyu Wang,[*,†] Miriam Marqués,[‡] Sergio Gómez,[¶,§] Francesc Serratosa,[¶] Eva Zurek,[‖] and Julia Contreras-García[*,†]

†*Sorbonne Université, CNRS, Laboratoire de Chimie Théorique, LCT, 75005 Paris, France*

‡*CSEC, School of Physics and Astronomy, University of Edinburgh, Edinburgh, EH9 3JZ, United Kingdom*

¶*Departament d'Enginyeria Informàtica i Matemàtiques, Universitat Rovira i Virgili, 43007 Tarragona, Spain*

§*ComSCIAM, Universitat Rovira i Virgili, 43007 Tarragona, Spain*

‖*Department of Chemistry, State University of New York at Buffalo, Buffalo, NY 14260-3000, USA*

E-mail: xiaoyu.wang@sorbonne-universite.fr; julia.contreras_garcia@sorbonne-universite.fr

## Contents

**S1 Test on Convolutional Neural Network** **3**

**S2 Convergence Tests of Hyperparameters** **5**

**S3 Extra Residue Analysis** **7**

**S4 Advanced Error Analysis** **8**

**References** **10**

# S1 Test on Convolutional Neural Network

We have additionally tested a single-channel three-dimensional convolutional neural network[1] (3D CNN) as an alternative model architecture to assess whether non-local correlations can be captured within a grid-based framework. For each sampling point $\mathbf{r}_0$, a local density patch is constructed as

$$\rho(\mathbf{x}) = \sum_{i \in r_{\text{cut}}} \exp\left(-\frac{|\mathbf{x} - (\mathbf{R}_i - \mathbf{r}_0)|^2}{2\sigma^2}\right), \tag{1}$$

where the sum runs over hydrogen atoms within a cutoff radius $r_{\text{cut}}$. The density is discretized on a cubic grid spanning $[-r_{\text{cut}}, r_{\text{cut}}]^3$.

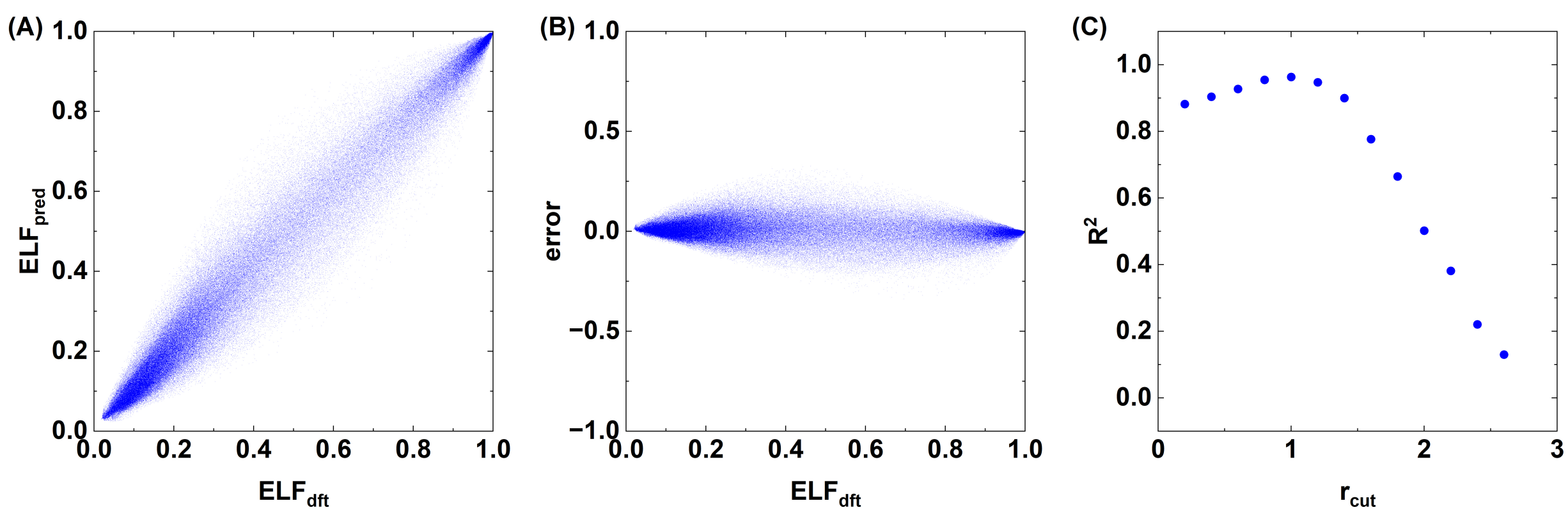


Figure S1: (A) Correlation between predicted and reference ELF values; (B) Prediction error ($\text{ELF}_{\text{pred}} - \text{ELF}_{\text{true}}$) as a function of the reference ELF; (C) Dependence of the coefficient of determination ($R^2$) on the cutoff radius $r_{\text{cut}}$.

The resulting input tensor is

$$\mathbf{X} \in \mathbb{R}^{N \times P^3}, \tag{2}$$

where $N$ is the number of sampled grid points and $P$ is the number of discretization points per spatial dimension. In this work, we set $P = 12$ and used a CNN consisting of three convolutional blocks followed by a sigmoid output layer. Using the same training protocol as for the MLP model[2] (50,000 samples per MD snapshot), we systematically varied the cutoff radius $r_{\text{cut}}$ from 0.25 to 2.5 Å. The best performance was obtained at $r_{\text{cut}} = 1.0$ Å,

yielding a maximum coefficient of determination $R^2 = 0.966$ for the 150,000 evaluation set.

Despite its increased representational flexibility, the CNN model exhibits several practical limitations. First, the input dimensionality becomes prohibitively large (on the order of $10^8$ elements for the present setup), which severely restricts model scalability. Second, the grid-based representation is inherently tied to a three-dimensional voxelization and is therefore not directly transferable to lower-dimensional analyses (e.g., line scans or planar slices), which are commonly used in ELF studies.

Importantly, the spatial structure of the prediction residuals remains qualitatively similar to that obtained with the MLP model, albeit with a broader distribution. In particular, the residual retains a pronounced long-wavelength character, indicating that the dominant source of error is not specific to the choice of model architecture. We further observe that increasing $r_{\text{cut}}$ does not improve performance beyond the optimal value. While a larger cutoff formally incorporates more long-range information, it also dilutes the resolution of short-range features due to the fixed grid size, leading to a decrease in predictive accuracy. This trade-off results in a clear optimum at $r_{\text{cut}} = 1.0$ Å. Overall, these results suggest that the observed long-wavelength residual is not primarily a consequence of the specific local regression model, but rather reflects intrinsic limitations in representing long-range correlations within a strictly local descriptor framework.

## S2 Convergence Tests of Hyperparameters

We performed systematic convergence tests with respect to the key hyperparameters of the model. Unless otherwise specified, the baseline configuration consists of a network width of 128, two hidden layers, a cutoff radius $r_{\mathrm{cut}} = 3.0$, $n_{\mathrm{radial}} = 10$, $l_{\mathrm{max}} = 2$, and a Huber loss parameter $\delta = 0.03$, trained for 80 epochs.

To ensure a consistent comparison across different hyperparameter settings, the random seeds used to generate the training set ($50{,}000 \times 3$ samples) and the evaluation set ($150{,}000 \times 3$ samples) were kept fixed throughout all tests.

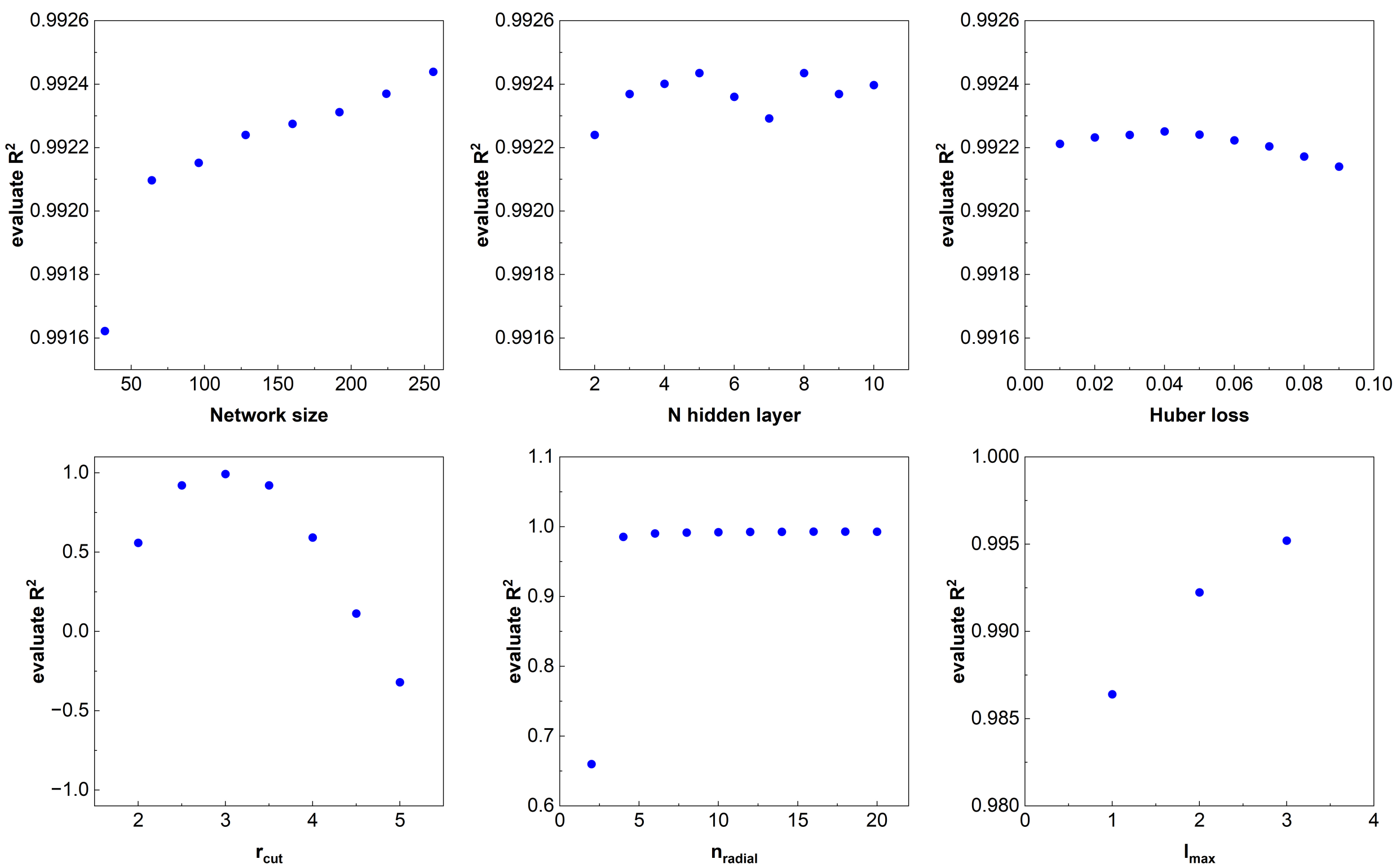


Figure S2: Convergence of model performance with respect to key hyperparameters. The coefficient of determination ($R^2$) on the validation set is shown as a function of (i) network width, (ii) number of hidden layers, (iii) Huber loss parameter, (iv) cutoff radius $r_{\mathrm{cut}}$, (v) number of radial basis functions $n_{\mathrm{radial}}$, and (vi) maximum angular momentum $l_{\mathrm{max}}$.

The impact of numerical precision was assessed by repeating the evaluation using float32 storage, yielding differences in validation $R^2$ below $10^{-5}$, indicating that float16 precision does not introduce measurable artifacts.

For a representative 500-atom hydrogen structure on a $192^3$ grid, the ML pipeline (descriptor construction and inference) requires approximately 5–8 seconds on a single NVIDIA V100 GPU.

# S3 Extra Residue Analysis

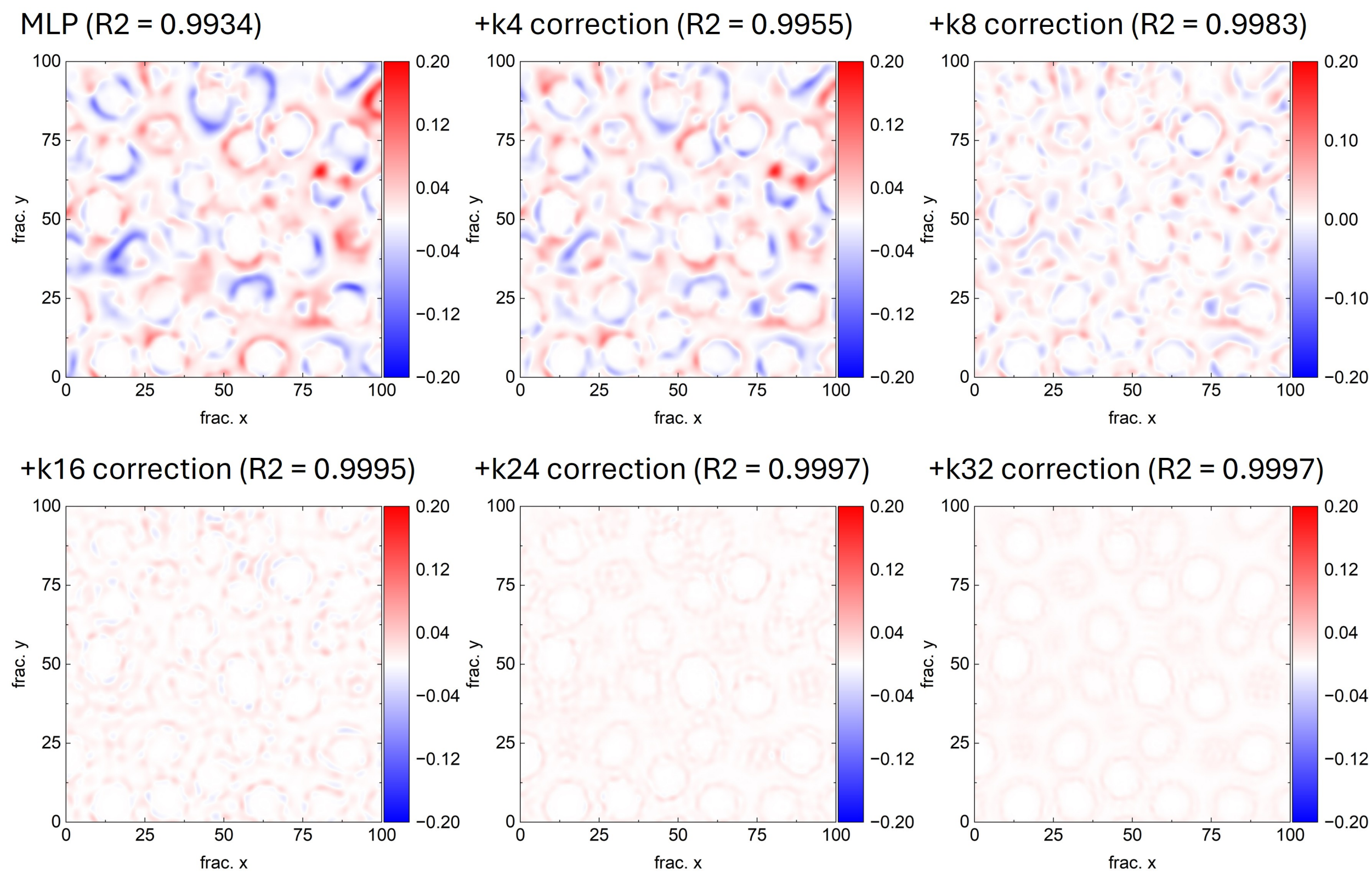


Figure S3: Real-space residuals between predicted and DFT ELF values for a representative two-dimensional slice of the 76.0 GPa structure. The color scale ranges from $-0.2$ (blue), through 0 (white), to $+0.2$ (red). Results are shown for the baseline model and for predictions after applying different $k$-correction schemes, in which the residual in logit space is projected onto a truncated low-$k$ Fourier basis to restore long-wavelength contributions beyond the local descriptor.

# S4 Advanced Error Analysis

To quantify the uncertainty of the reported metrics, we repeated the entire workflow—including training set generation, model training, and validation set sampling—50 times using different random seeds, resulting in an ensemble of 50 independently trained models. For ELF training and validation, the resulting standard deviations of MAE, RMSE, and $R^2$ are on the order of $10^{-5}$, indicating negligible stochastic uncertainty and highly stable model performance.

The same ensemble was then applied to ELF prediction and networking value (NW) prediction on randomly generated structures using RandSpg.[3] In this case, the global standard deviation across all structures is significantly larger; however, this spread is dominated by genuine variation in performance among different structures rather than training stochasticity. Accordingly, we analyze the results on a per-structure basis, where the run-to-run variability remains small and the variation of the structure-wise mean values reflects differences in structural complexity. All individual metrics for the 50 runs, including both ELF and NW predictions, are provided in an accompanying Excel file for completeness.

Table S1: Mean and standard deviation of ELF prediction metrics and networking value (NW) predictions for training data and randomly generated (RandSpg) cubic and hexagonal hydrogen structures. Statistics are computed from an ensemble of 50 independently trained models and reported globally (across all samples).

| | mean MAE | stdev | mean RMSE | stdev | mean $R^2$ | stdev |
|---|---|---|---|---|---|---|
| Training | | | | | | |
| MLP | 0.0190 | $6.83 \times 10^{-5}$ | 0.0271 | $9.06 \times 10^{-5}$ | 0.992 | $5.39 \times 10^{-5}$ |
| RandSpg (ELF) | | | | | | |
| Cubic | 0.0385 | 0.0159 | 0.0520 | 0.0196 | 0.956 | 0.0421 |
| Hexagonal | 0.0470 | 0.0148 | 0.0635 | 0.0200 | 0.947 | 0.0407 |
| RandSpg (NW) | | | | | | |
| Cubic | 0.0331 | 0.0243 | 0.0372 | 0.0246 | 0.940 | - |
| Hexagonal | 0.0459 | 0.0305 | 0.0506 | 0.0304 | 0.866 | - |

To further validate the model transferability, we performed an additional test on a completely independent set of snapshots taken from AIMD trajectories. At each pressure point

(76.0, 115.1, and 138.5 GPa), two snapshots were selected at 1000 and 2000 MD steps, corresponding to 0.5 and 1.0 ps after equilibration. Following the same procedure as in the main manuscript, 150,000 random voxels were sampled from each snapshot. The model was not retrained; instead, the same model used for the production calculations in the manuscript was directly applied. The results are summarized in Table S2

Table S2: Prediction performance (MAE, RMSE, and $R^2$) for independent AIMD snapshots (1000 and 2000 steps; 0.5 and 1.0 ps after equilibration) at each pressure point.

| Pres. | Snapshot | MAE | RMSE | $R^2$ |
|---|---|---|---|---|
| 76.0 | 1000 | 0.0194 | 0.0265 | 0.991 |
| 76.0 | 2000 | 0.0197 | 0.0268 | 0.991 |
| 115.1 | 1000 | 0.0216 | 0.0305 | 0.991 |
| 115.1 | 1000 | 0.0211 | 0.0298 | 0.991 |
| 138.5 | 1000 | 0.0185 | 0.0279 | 0.993 |
| 138.5 | 2000 | 0.0185 | 0.0279 | 0.993 |

# References


(1) LeCun, Y.; Bottou, L.; Bengio, Y.; Haffner, P. Gradient-based learning applied to document recognition. *Proc. IEEE* **2002**, *86*, 2278–2324.

(2) Hornik, K.; Stinchcombe, M.; White, H. Multilayer feedforward networks are universal approximators. *Neural Netw.* **1989**, *2*, 359–366.

(3) Avery, P.; Zurek, E. RandSpg: An open-source program for generating atomistic crystal structures with specific spacegroups. *Comput. Phys. Commun.* **2017**, *213*, 208–216.
